# Accuracy Analysis of Feature-Based Automatic Modulation Classification via Deep Neural Network

**DOI:** 10.3390/s21248252

**Published:** 2021-12-10

**Authors:** Zhan Ge, Hongyu Jiang, Youwei Guo, Jie Zhou

**Affiliations:** Institute of Electronic Engineering, China Academy of Engineering Physics, Mianyang 621000, China; gezhan@caep.cn (Z.G.); guoyouwei@caep.cn (Y.G.); zhoujie_iee@caep.cn (J.Z.)

**Keywords:** automatic modulation classification, deep learning, higher-order cumulants, fuzzy c-means clustering, non-Gaussian channel, flat-fading

## Abstract

A feature-based automatic modulation classification (FB-AMC) algorithm has been widely investigated because of its better performance and lower complexity. In this study, a deep learning model was designed to analyze the classification performance of FB-AMC among the most commonly used features, including higher-order cumulants (HOC), features-based fuzzy c-means clustering (FCM), grid-like constellation diagram (GCD), cumulative distribution function (CDF), and raw IQ data. A novel end-to-end modulation classifier based on deep learning, named CCT classifier, which can automatically identify unknown modulation schemes from extracted features using a general architecture, was proposed. Features except GCD are first converted into two-dimensional representations. Then, each feature is fed into the CCT classifier for modulation classification. In addition, Gaussian channel, phase offset, frequency offset, non-Gaussian channel, and flat-fading channel are also introduced to compare the performance of different features. Additionally, transfer learning is introduced to reduce training time. Experimental results showed that the features HOC, raw IQ data, and GCD obtained better classification performance than CDF and FCM under Gaussian channel, while CDF and FCM were less sensitive to the given phase offset and frequency offset. Moreover, CDF was an effective feature for AMC under non-Gaussian and flat-fading channels, and the raw IQ data can be applied to different channels’ conditions. Finally, it showed that compared with the existing CNN and K-S classifiers, the proposed CCT classifier significantly improved the classification performance for MQAM at N = 512, reaching about 3.2% and 2.1% under Gaussian channel, respectively.

## 1. Introduction

Automatic modulation classification (AMC) determines the underlying modulation type of intercepted signals from a given set of modulation schemes [1]. It plays an important role in many fields, such as cognitive radio, software-defined radio, interference identification, and spectrum management. Over the years, issues of AMC have been sufficiently studied in the literature. However, it remains a challenging task in some non-ideal channel environments. Most existing AMC algorithms can be grouped into two categories, namely, likelihood-based (LB) and feature-based (FB) algorithms [2,3]. The LB algorithm usually treats AMC as a problem of multiple hypothesis testing. It always suffers from high computational complexity, although it is the optimal classifier in the Bayesian sense. Meanwhile, it also requires the perfect knowledge of channel state information (CSI), which is not always available in practice [4,5,6]. On the other hand, the FB algorithm usually provides sub-optimal solutions. However, it can be executed efficiently with lower computational complexity compared with the previous algorithm [7]. In addition, it does not rely on the prior knowledge of CSI. When the distinctive features are well designed, it can provide better classification performance under most channel conditions [8]. As a result, the FB algorithm has been sufficiently investigated and applied under various scenarios.

The FB-AMC usually consists of two key components: features’ extraction and classifiers. Features’ extraction component is used to calculate distinguishing features from received signals. Then, these features will be fed to classifiers to determine the modulation formats. In the literature, numerous features have been proposed for modulation classification so far. The most frequently used features for FB-AMC include instantaneous statistics [9], high-order signal statistics [10,11], cyclic spectrum [12,13], wavelet transform [14], cyclic-cumulant [15,16,17], constellation diagram [18,19,20], cumulative distribution function [21,22,23,24,25], time-frequency features [26], and so on. Accordingly, several machine learning methods have been extensively studied as classifiers for FB-AMC, such as decision trees, support vector machines (SVM), K-nearest neighbors (KNN), artificial neural networks (ANN), and clustering algorithms [9,10,11,12,13,14,27,28].

FB-AMC is quite effective and robust in some scenarios. However, most FB methods make classification decisions on the threshold of the extracted features, which always heavily rely on expert knowledge [9,10,11]. Once the features’ and decisions’ thresholds are not well designed, the AMC cannot be performed well. Especially under non-ideal channel conditions, it is not easy to obtain distinctive features for classification without channel estimation [10]. In this case, an ideal algorithm that can directly learn the discriminative representations from the input is needed. On the other hand, traditional machine learning methods are commonly used classifiers and achieve better performance over the years. However, these methods have low efficiency when processing large-scale data and cannot handle data samples with uneven distributions. For example, it is difficult to use traditional machine learning methods to learn the features of constellation diagrams [19]. Recently, the literature showed that deep learning methods can automatically and directly learn distinguishing features from the inputs to accomplish AMC without manually designing decisions’ threshold [29,30]. Additionally, deep learning models can take large-scale complex data as input, which is suitable for processing image features. Therefore, it provides a great example to classify the unknown modulation scheme of received signals by combining deep learning methods and extracted features.

Deep learning is a fast-growing branch of machine learning and has achieved promising successes in various engineering fields, such as image processing, computer vision (CV), natural language processing (NLP), and object detection [31]. The commonly used DL architectures include autoencoders (AE) [32,33], convolutional neural network (CNN) [34,35,36,37], long short-term memory (LSTM) [38,39,40,41,42], generative adversarial networks (GAN) [43], and deep Q-learning algorithm (DQN) [44]. Due to their excellent capabilities in the above fields, deep learning models have been extended to the field of automatic modulation classifications. Reference [30] proposed a novel modulation classification method based on fourth-order cumulants and a k-sparse autoencoder with a non-negativity penalty. Compared with the SVM classifier, this method achieves better performance with lower computational complexity. In [19], received signals were projected into constellation diagrams, and two CNN-based models (i.e., AlexNet and GoogLeNet) were adopted to explore high-level features from the constellation diagrams for further classification. Due to the superior image recognition ability of the CNN networks, classification accuracy in [19] was close to the optimal performance. Several CNN-based methods were proposed in [34,35,36], which can automatically accomplish modulation classification directly from complex received signals without manually designing features. However, most of the above studies only considered one channel condition or one feature for AMC.

In this study, we aimed to compare the performance of extracted features for FB-AMC by using a deep learning method to identify M-PSK and M-QAM modulation schemes under various channels. A deep neural network (DNN)-based method (CCT classifier) was proposed for FB-AMC. Several training strategies, e.g., early stopping and scheduler learning rate, were introduced into the network to improve classification accuracy. In order to guarantee a fair comparison, the deep network for each feature was trained individually according to different channel conditions to gain the best performance. It is well known that most automatic modulation classification algorithms in the literature assume that the channel noise is Gaussian additive noise [1]. However, it is also shown that most communication channels experience fading, non-Gaussian noise, interference, etc. [45,46]. These impairments of channels will degrade the accuracy of the methods based on Gaussian additive noise [47,48,49]. To consider a realistic channel environment, Gaussian channel (AWGN), phase offset, frequency offset, non-Gaussian channel, and flat-fading channel were introduced to evaluate the classification accuracy of different features.

The main contributions of this study are summarized as follows.

1. We propose an end-to-end modulation classifier for automatic modulation classification problems under three channel conditions, including Gaussian, non-Gaussian, and flat-fading channels. Five different features, including HOC, features-based fuzzy c-means clustering (FCM), GCD, CDF, and raw IQ data, were used for the comparative study. To the best of our knowledge, few works have considered these channel conditions and features at the same time. We are confident that this study of FB-AMC using classical features and deep learning methods will be beneficial to further work on automatic modulation classification.
2. The proposed CCT classifier composed of CNN-2D, CNN-1D, and TCN can handle variable inputs with different shapes. The lightweight networks CNN-1D and TCN were used to extract spatial and temporal information to improve training efficiency and modulation classification performance.
3. Since extensive experiments were performed in this study, transfer learning was introduced to reduce the time cost of the training process. Results showed that the training accuracy and training loss were improved efficiently for a new dataset with the help of transfer learning.
4. Experiments’ results showed that, compared with the traditional K-S classifier and one existing CNN classifier, the proposed CCT classifier obtained better performance.

The rest of this paper is organized as follows. In Section 2, signal models under Gaussian, flat-fading, and non-Gaussian channels and different features are presented. Section 3 describes the general architecture of the CCT classifier proposed for the comparative study of FB-AMC. Section 4 is about experimental results. Section 5 analyzes the experimental results and discusses future work. Finally, the conclusion is drawn in Section 6.

## 2. Materials

### 2.1. System Model

The general representation of modulated signals after matched filtering is given as follows:

(1) $$r\left(n\right)=\alpha e^{j\left(2\pi f_{0}n+\theta_{0}\right)}s\left(n\right)+\omega \left(n\right)$$

where α represents the attenuation factor, $\theta_{0}$ is the phase offset, $f_{0}$ is the frequency offset, $\omega \left(n\right)$ is the additive noise, and n is the symbol index. The transmitted symbols $s\left(n\right)$ are drawn from the predetermined set of modulation schemes with equal probability and are assumed to be independent. Without loss of generality, the energy of transmitted symbols $E{\left\{\left|s\left(n\right)\right|\right\}}^{2}$ is normalized to unity.

In this study, to make a comparative study of FB-AMC under different channel conditions, besides the Gaussian channel, non-Gaussian and flat-fading channels were also considered.

Gaussian channel: Assume that α  = 1 and $\omega \left(n\right)$ is complex additive white Gaussian noise, which follows the distribution $CN\sim \left(0,\;\sigma^{2}\right)$. The SNR (signal-to-noise ratio) is defined as the ratio of the averaged received signals’ power to the noise variance. Therefore, the SNR is equal to $10log10\left(1/\sigma^{2}\right)$.

Non-Gaussian channel: In this case, assume that α = 1 and the phase offset and frequency offset are not considered. The additive noise $\omega \left(n\right)$ is assumed to follow the N-term Gaussian mixture model (GMM). The probability density function (PDF) of $\omega \left(n\right)$ is shown as follows:

(2) $$p\left(\omega \right)=\sum_{n=1}^{N}\frac{\lambda_{n}}{2\pi \sigma_{n}^{2}}\exp\left(-\frac{{\left|\omega \right|}^{2}}{2\sigma_{n}^{2}}\right)$$

where $\lambda_{n}$ denotes the probability that the Gaussian noise $\omega \left(n\right)$ is drawn from the n-th component in the PDF, which satisfies $0<\lambda_{n}<1,$ and $\sum_{n=1}^{N}\lambda_{n}=1$, $\sigma_{n}^{2}$ is the energy of the n-th component [47,48]. The SNR is defined as the ratio of the averaged received signals’ power to the 1-th component Gaussian noise variance, i.e., $10log10\left(1/2\sigma_{1}^{2}\right)$.

Flat-fading channel: Here, the channel is treated as a flat-fading channel. It means that the α, $\theta_{0}$, and $f_{0}$ are assumed to be constants in each received signal samples over the observation period. The $\omega \left(n\right)$ is complex additive white Gaussian noise. The signal model for this case can be expressed as:

(3) $$r\left(n\right)=\alpha s\left(n\right)+\omega \left(n\right)$$

where α represents not only the flat-fading experienced by the signal and unknown power but also the carrier phase of the transmitted signal [45,46]. The amplitude of α is assumed to be Rayleigh distributed, with $E\left({\left|\alpha \right|}^{2}\right)=1$, and the phase of α is uniformly distributed in $\left(0,\;2\pi \right]$. The received SNR is the same as that of the Gaussian channel, i.e., $10log10\left(1/\sigma^{2}\right)$.

### 2.2. Features’ Extraction

In this subsection, we describe the extraction of various features used in this comparative study, including higher-order cumulants (HOC), features-based fuzzy c-means clustering (denoted “FCM” in this paper), grid-like constellation diagram (GCD), cumulative distribution function (CDF), and raw IQ data. The end-to-end deep neural network classifier (CCT classifier) developed to address problems in FB-AMC is described in Section 3 in detail.

#### 2.2.1. Higher-Order Cumulants (HOC)

Higher-order cumulants are popular and low-complexity features to automatic modulation classification for their better performance of anti-noise and anti-inference [10]. In [10], simulation results showed that the distribution shape of noisy signal constellations can be characterized by HOCs. Over the years, the fourth-order, sixth-order, and eighth-order cumulants have been proposed in the literature [33,46]. It has been proven that the combination of different order cumulants can improve classification accuracy. In addition, it should be noted that the eighth-order cumulants can be utilized to distinguish high-order modulation schemes, but the increased computational complexity should be considered. Therefore, we chose the fourth- and sixth-order cumulants of $r\left(n\right)$ as features for the classification of MPSK and MQAM signals in this study.

Given N signals’ samples $\left\{r{\left(n\right)}_{n=1}^{N}\right\}$, the mixed moments can be obtained by

(4) $$M_{km}=E\left[r^{k-m}{\left(r^{*}\right)}^{m}\right]$$

HOCs of various orders are given by the following equation. For details, please refer to [10,11,37].

(5) $$C_{km}\left(x\right)=Cum\left\{r^{k-m}{\left(r^{*}\right)}^{m}\right\}$$

In addition, assuming that the energy of signals is normalized to unity, theoretical values of fourth-order and sixth-order cumulants of different modulation schemes under ideal conditions are shown in Table 1.

#### 2.2.2. Grid-like Constellation Diagrams (GCDs)

For MQAM and MPSK signals, they can be characterized by their constellation diagrams (CDs). Motivated by the fact that deep learning methods are specialized in image processing, it is intuitive to turn the AMC problem into an image recognition problem [18,19,20]. Generally, constellation diagrams generated by plotting the real parts and image parts of the complex signals are gray images with only one channel information. It cannot reflect the density of constellation points since multiple points are aggregated in one pixel. In this paper, a data conversion algorithm proposed in [20] was introduced to convert the CDs into three-channel images with density information of constellation points, named grid-like constellation diagrams (GCDs). In addition, we used a Gaussian filter to smooth the density of constellation points. The highlighted region in GCDs implies that there are multiple data points in this region.

**Figure 1:**
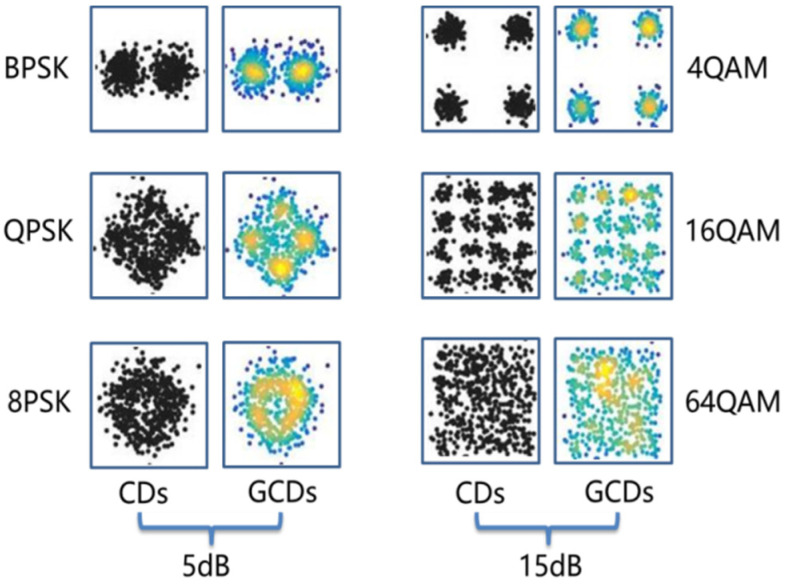
Grid-like constellation diagrams of MPSK (**left**) and MQAM (**right**).

GCDs of MPSK (2PSK, 4PSK, 8PSK) at SNR = 5 dB and MQAM (4QAM, 16QAM, 64QAM) at SNR = 15 dB under the Gaussian channel are illustrated in Figure 1. It can be observed that GCDs have more discernible information than CDs. Therefore, the main idea of FB-AMC based on constellation diagrams is to convert received signals to GCDs firstly, and then a deep neural network is adopted to learn from the GCDs for further classification.

#### 2.2.3. Features-Based Fuzzy c-Means Clustering (FCM)

Clustering is an unsupervised method that allows each observation to belong to multiple clusters. The commonly used clustering algorithms include k-means, subtract clustering, FCM, GMM, etc. [50,51,52,53]. Modulation signals characterized by constellation shapes, such as MPSK and MQAM, can be classified by clustering algorithms. In this paper, we utilize FCM clustering to reconstruct the constellation diagrams of the received signals. Then, we extract the discriminating features by the mean hard tendency of fuzzy clustering proposed in [53]. Finally, the clustering features are sent to the deep neural network to recognize the modulation scheme of received signals.

The mean hard tendency is a clustering validity measure to evaluate the performance of FCM clustering. It can be performed by the following steps.

1. Given the number of clusters D, for each constellation data point $x_{j}$, define a relation between the second $u_{kj}$ and the first maxima $u_{ij}$ of elements in $U_{\cdot j}$. The relation indicates that the data $x_{j}$ how hard ($r_{j}\to 0$) or how fuzzy ($r_{j}\to 1$) belongs to the given d-partition.
  (6) $$r_{j}=\frac{u_{kj}}{u_{ij}},\;\;\;\;\;u_{ij}=\underset{0<t<D}{\max}\left\{u_{tj}\right\},\;\;\;\;u_{kj}=\underset{0<t<D,t\neq i}{\max}\left\{u_{tj}\right\}$$
2. Translate fuzzy d-partition into a hard partition by assigning the point $x_{j}$ to the cluster with the maximum membership function.
  (7) $$Y_{i}=\left\{x_{j}/\mu_{ij}=\underset{0\leq t\leq d}{\max}\{U_{tj}\right\},\;\;\;x_{j}\in X$$
3. Define the hard tendency of *i*-th cluster by calculating the mean of all $x_{j}$ with the data points that belong to the *i*-th cluster in the hard partition $Y_{i}$.
  (8) $$T_{D_{i}}=\frac{1}{N_{i}}\sum_{x_{j}\in Y}r_{j}$$
4. The mean hard tendency is defined as the average of $T_{D_{i}}$
  (9) $$T_{D}=\frac{1}{D}\sum_{i=1}^{D}-\log\left(T_{D_{i}}\right)$$

**Figure 2:**
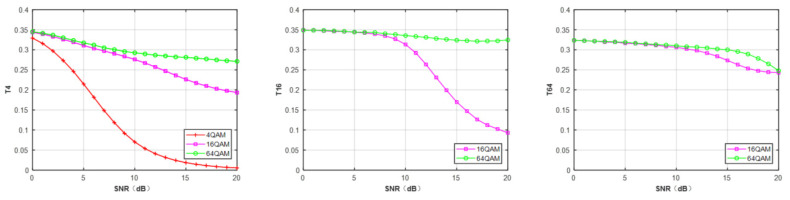
The mean hard tendency of $T_{D}$ of MQAM at N = 512.

In this paper, the number of clusters D is set to 2, 4, and 8 for MPSK and 4, 8, and 16 for MQAM. Figure 2 illustrates the features $T_{D}$ of three QAM signals calculated under different clusters’ number D. In Figure 2, we observe that different modulated signals have a discriminating value of $T_{D}$. Thus, the FCM-based features, $T_{D}$ can be adopted as the inputs of deep neural networks to classify the modulation schemes.

#### 2.2.4. Cumulative Distribution Function (CDF)

The Kolmogorov–Smirnov test classifier (K–S classifier) proposed in [21] is performed by comparing the distance between the theoretical cumulative distribution function (CDFs) and the empirical cumulative distribution function (ECDFs) of received signals [22,23,24,25]. Different from the K–S classifier, which aims to find the minimum value from the set of maximum distances between ECDFs and CDFs calculated for each modulation scheme, our proposed method uses a deep neural network to learn high-level representations from ECDFs without calculating the CDFs again. In addition, the K–S classifier only considers one test point with the maximum distance, while our method can explore the distinct information within all the test points.

This paper calculates the ECDF of MQAM and MPSK, respectively. For QAM signals, the modulation information is mainly represented by magnitude. Therefore, the magnitude of QAM signals can be defined as decision statistics, i.e.,

(10) $$z_{n}=\left|r_{n}\right|=\sqrt{ℜ{\left\{r_{n}\right\}}^{2}+ℑ{\left\{r_{n}\right\}}^{2}},\;\;\;\;n=1,\;2,\ldots ,\;N$$

For PSK signals, the modulation information is represented by phase. Therefore, the phase of MPSK signals is defined as decision statistics, i.e.,

(11) $$z_{n}=\arctan\left(\frac{ℑ\left(r_{n}\right)}{ℜ\left(r_{n}\right)}\right)$$

Then, the ECDFs of decision statistics is calculated by:

(12) $${\hat{F}}_{1}\left(z\right)=\frac{1}{N}\sum_{n=1}^{N}I(z_{n}<z)$$

where $I\left(\cdot \right)$ is the indicator function, which equals 1 if $z_{n}<z$ or equals to 0 otherwise. N is the length of signals.

**Figure 3:**
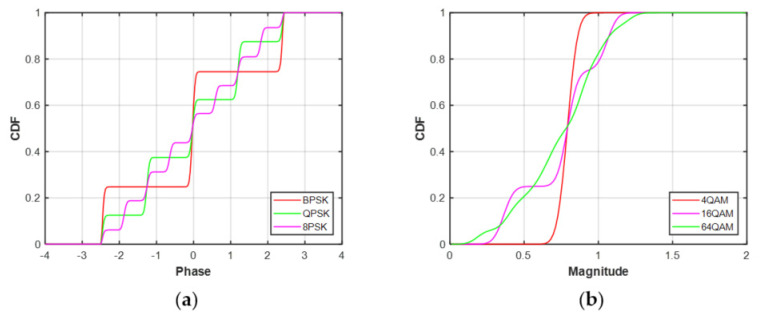
The ECDFs of decision statistics at SNR = 20 dB, N = 128. (**a**) MPSK. (**b**) MQAM.

The ECDFs of MPSK and MQAM signals at SNR = 20 dB and N = 128 are shown in Figure 3. It can be seen that there are obvious differences in the ECDFs of the MPSK phase distribution. Similarly, the ECDFs of MQAM amplitude distribution are also significantly different. Hence, the modulation classification of MPSK and MQAM can be performed by feeding the ECDFs into the deep neural network, respectively.

#### 2.2.5. Raw IQ Data

Recently, some references have shown that deep learning methods have a powerful capability to learn discriminating information directly from received signals to classify modulation schemes without calculating features in advance [34,35,36,37,38,39,40,41]. However, the preprocessed received signals are always complex data composed of real parts (I components) and imaginary parts (Q components). These complex data are difficult to be directly trained by a deep neural network because the weights and biases of neural networks need to be real values. Therefore, the received complex signals should be processed before being fed into a deep neural network. In this paper, we re-represent the complex received signals by separating the I and Q components into $ℜ\left(\cdot \right)$ and $ℑ\left(\cdot \right)$ firstly, and then rearranging them into two-dimensional matrix data by (13). The processed data are titled raw IQ data and are treated as a feature here. They contain all the amplitude and phase information of received signals. For example, received complex signals $r=\left[r\left(0\right),r\left(1\right),\cdots r\left(N-1\right)\right]$ can be presented as, i.e.,

(13) $$R={\left[\begin{array}{cccc} ℜ\left(r\left(0\right)\right) & ℜ\left(r\left(1\right)\right) & \cdots & ℜ\left(r\left(N-1\right)\right)\\ ℑ\left(r\left(1\right)\right) & ℑ\left(r\left(1\right)\right) & \cdots & ℑ\left(r\left(N-1\right)\right) \end{array}\right]}^{T}$$

where, $ℜ\left(\cdot \right)$ and $ℑ\left(\cdot \right)$ represent the real parts and imaginary parts of received signals, respectively. Then, the two-dimensional data are used as training data of the deep neural network for modulation classification.

To conclude, the above five features can be used to accomplish the problems of FB-AMC. However, each feature has a different shape, as shown in Table 2. For example, the HOCs include nine sub-features ($C_{20},\;C_{21},\;C_{40},C_{41},\;C_{42},\;C_{60},\;C_{61},\;C_{62},\;C_{63}$) with a shape 1 × 9, while GCDs are three-channel images with a shape 64 × 64 × 3. Thus, how to deal with the features of different shapes to meet the input format of the deep neural network is a problem. It will be described in Section 3.1.

## 3. Method

In this Section, we develop a novel end-to-end modulation classifier based on deep neural networks to classify MPSK and MQAM signals by analyzing the features extracted from received signals. The proposed CCT classifier comprised of two-dimensional CNN, one-dimensional CNN, and temporal convolutional network (CNN-2D, CNN-1D, and TCN) was designed to process the different features in a general architecture so that we would not need to design a new deep neural network for each feature. The architecture of the deep CCT classifier is presented in detail in Section 3.1. In addition, the ideal maximum likelihood (ML) classifier is described in Section 3.3, which provides an upper bound of classification performance for the CCT classifier under ideal/non-ideal channel conditions.

### 3.1. The Architecture of CCT Classifier

**Figure 4:**
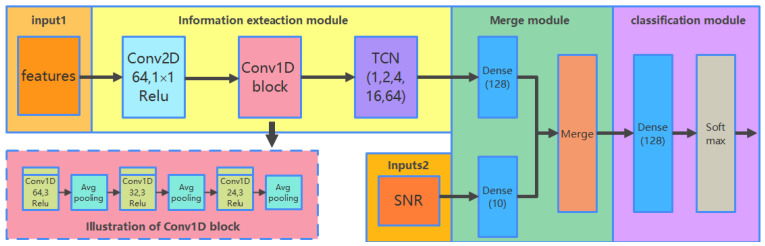
Structure of the proposed CCT modulation classifier.

The CCT classifier identifies the modulation schemes of received signals by exploring deep representations of different features. As shown in Figure 4, the framework of the CCT classifier consists of four components: inputs, information extraction module, merging module, and classification module.

The inputs of CCT consist of two parts: extracted features and estimated symbols SNR. In this paper, we aim to compare the performance of modulation classification in the extracted features with the different shapes listed in Table 2. For a fair comparison, the proposed classifier should have the capability to deal with inputs in different formats. Therefore, inputs of features are transformed into the same format. Another scalar input of estimated SNR is processed by a full-connect neural network.

The fundamental component of CCT, called the information extraction module, is formed by cascading three types of neural networks, CNN-2D, CNN-1D, and TCN. CNN-2D network is regarded as a “transition layer” with only one two-dimensional (2-D) convolutional layer. It is responsible for extracting information from the 2-D inputs and reshaping the outputs to a one-dimensional (1-D) vector. The lightweight CNN-1D block with fewer training parameters is followed to extract the spatial information from the outputs of CNN-2D for faster implementation [36]. TCN is followed to extract temporal information for more discriminative information to improve the performance of AMC. In the proposed network, the dilated factors of TCN were set to 1, 2, 4, 16, and 64, respectively. The kernel filter size was set to 3. The merging and classification module was used to concatenate the outputs and accomplish the classification.

Inputs: In Table 2, it is observed that GCDs are three-channel images and the raw IQ data are a 2-D matrix data. They are always processed by a two-dimensional CNN, which has the initially well-known ability to learn from images [34]. Correspondingly, the features of HOC, FCM, and CDF are 1-D sequence data, which are usually suitable for being processed with one-dimensional CNN. In order to make the features of different shapes satisfy the input format of the proposed deep classifier, we made a trade-off between these features. According to the representation format of raw IQ data, features except for GCD were transformed into 2×N two-dimensional matrix representations, where N is the length of received signals. It should be noted that the lengths of HOCs and FCM were 9 and 3, which were much shorter than the received signal length N. Therefore, to fill the transformed 2-D matrix, they were repeated N/N1 times in each row (N1 is the length of the feature), and the blank spaces of the matrix were filled with zeros. After data conversion, all features were processed by the CNN-2D network. The outputs of extracted features and estimated symbols SNR were then concatenated in the merging and classification module for AMC.

For example, the feature of FCM $\left[T_{2},\;T_{4},\;T_{8}\right]$ will be converted into the following format:

(14) $${\left[\begin{array}{ccccccccc} T_{2} & T_{4} & T_{8} & \cdots & T_{2} & T_{4} & T_{8} & \cdots & 0\\ T_{2} & T_{4} & T_{8} & \cdots & T_{2} & T_{4} & T_{8} & \cdots & 0 \end{array}\right]}_{2\times N}$$

CNN-2D: Note that “Conv2D 64, 1 × 1” in Figure 4 denotes that the 2-D convolution layer has 64 channels with a 1 × 1 convolution filter. To make full use of the underlying information of inputs, the filters stride step was set to one. Mathematically, the convolutional operation is represented as,

(15) $$x_{j}^{l}=f(\sum_{i\in M_{j}}x_{i}^{l-1}\times k_{ij}^{l}+b_{j}^{l})$$

where $x_{j}^{l}$ represents the j-th feature maps of the l-layer, $k_{ij}^{l}$ denotes the convolutional kernel, $M_{j}$ is feature maps, and $f\left(\cdot \right)$ and $b_{j}^{l}$ are the activation function Relu and bias.

CNN-1D: This block compresses the outputs of CNN-2D into a 1-D temporal representative vector, which is easy to be trained by the following network. In Figure 4, the CNN-1D block contains three parts, where every two 1-D convolutional layers are followed by a 2 × 2 average pooling layer. There are nine layers in the block, including six 1-D convolutional layers and three 1-D average pooling layers. The convolutional operations of CNN-1D are similar to those in the CNN-2D block listed in Equation (15). Compared with the 2-D convolutional layer, the 1-D convolutional layer contains fewer parameters and strides through the vector with only one dimension. Therefore, it consumes less time during training. The average pooling layers are involved to compress the network parameters to reduce the computational complexity. Besides, the translation invariance of pooling layers will enhance sparsity to avoid overfitting when training the network. The pooling size was set to 2 in this paper. The operation of average pooling is represented as below.

(16) $$x_{k}^{l}=f\left(w_{j}^{l}pool\left(x_{j}^{l-1}\right)+b_{j}^{l}\right)$$

where $w_{j}^{l}$ and $b_{j}^{l}$ denote the weights and biases of pooling layers and $pool\left(\cdot \right)$ is the average pooling function.

**Figure 5:**
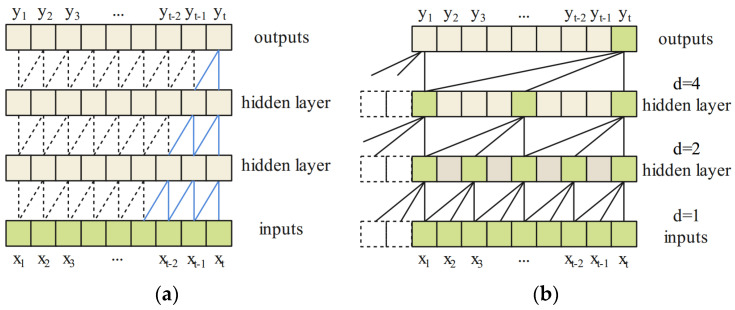
Illustration of casual convolution (**a**) and dilated casual convolution with dilated factors = 1, 2, and 4 and filter size = 3 (**b**).

TCN: TCN is based on CNN architecture. It usually consists of causal convolution, dilated convolution, and residual blocks [54,55]. TCN network just with the casual convolution is shown in Figure 5a. The step length and kernel interval are set to 1 here. Causal convolution aims to extract historical information before the current point and keep the length of outputs equal to the inputs. However, applying causal convolution to memorize long-term information from sequence data will cause the receptive field size to become limited. It will cause the vanishing or explosion of the gradient.

To solve this problem, dilated convolution was employed to obtain sufficiently large receptive fields by discarding some inputs with a given exponential dilated factor. TCN network with the dilated casual convolution is shown in Figure 5b. It is observed, that as the number of layers increase, the receptive fields grow exponentially. Therefore, more receptive fields will be obtained, which helps prevent the network from overfitting.

**Figure 6:**
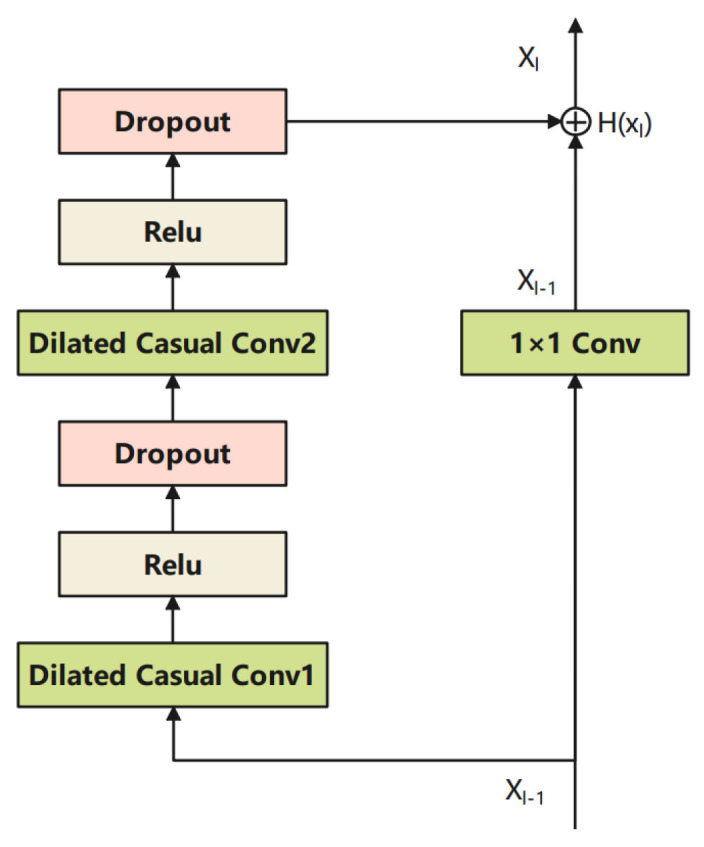
Illustration of the residual block.

In TCN, the residual block is also introduced to avoid vanishing gradient problems, as shown in Figure 6. The output of the previous layer $x_{l-1}$ is fed to the layer $x_{l}$ directly by an identity mapping. Even if the dilated casual convolution was learned badly, the performance of the residual block will be slightly affected. This strategy keeps the network away from the vanishing of the gradient, especially for deeper neural networks. The mathematical description of the residual structure is listed as follows.

(17) $$x_{l}=H_{l}\left(x_{l-1}\right)+x_{l-1}$$

where $x_{l}$ represent the outputs of layer l and $H\left(\cdot \right)$ denotes the output of the dilated causal convolution layer.

LSTM network is another effective module that can learn temporal features from sequence data. It shows that the LSTM network can significantly improve classification accuracy [39,41]. However, when learning from long sequence data, it will suffer from the problem of gradient vanishing and short memory length. In addition, LSTM uses the “Gate Mechanism” to learn historical information, which always causes parameters’ redundancy. Therefore, it will consume more time during training. Compared with LSTM, TCN can achieve similar performance but has lower computational complexity. It can obtain more historical information with few parameters increasing. Moreover, the 1-D convolution operations in TCN can be executed in parallel to accelerate the training process. Hence, in the CCT classifier, a TCN module is introduced to learn more high-level temporal information from the outputs of the CNN1D module to improve the classification accuracy of AMC.

Merging and classification module: The merging component is used to concatenate the outputs of TCN and the outputs of the estimated SNR. Then, the classification module including dense layers and a fully connected softmax layer is followed to predict the probability distribution of the merging outputs. The mathematical description of this module is listed as follows.

(18) $$predict=softmax\left(w_{l}\times x_{i}+b_{l}\right)$$

After the CCT network is built, it will be trained end-to-end according to different training datasets. During the training process, all parameters of the CCT network are updated by stochastic gradient descent (SGD) algorithm, with batch size set to 100 and learning rate set to 0.001 initially. Some strategies such as learning rate early stopping and scheduler learning rate are involved to improve the training performance of DNN. The number of iterations is set to 100, and it will stop early according to the validation loss.

### 3.2. Benefits of Applying CCT Classifier

The proposed CCT classifier composed of two-dimensional CNN, one-dimensional CNN, and TCN has three benefits to approach the problem of FB-AMC.

1. Flexibility of Variable Input Shape

Actually, it is difficult for a general deep framework to deal with inputs with different shapes. Most of the time, the framework of deep neural networks needs to be re-designed to handle different types of inputs. To address this problem, the extracted features, HOCs, FCM, and CDF, are transformed into 2-D matrix representations, which can be processed by a 2-D CNN. In this way, the CCT designed with a 2-D convolution layer as the first layer can learn high-level information from all features for classification. Therefore, the proposed CCT classifier can train and test variable inputs with different shapes based on the data transformation method.

2. Combination of Spatial and Temporal Information

Literature shows that 1-D CNN is beneficial in learning spatial features, while TCN is good at learning temporal features [39]. Inspired by this fact, the proposed CCT classifier uses CNN-1D to learn spatial information from the outputs of CNN-2D, and TCN is cascaded to summarize temporal information from the outputs of CNN-1D. The simulation results proved that the combination of these two networks can learn more discriminative features from the inputs, which helps to improve the performance of modulation classification.

3. Low Complexity of CCT classifier

In fact, the traditional 2-D CNN is good at handling image data. Therefore, radios’ signals are always converted into images at the beginning for further high-level information learning. However, modulated signals are usually considered to be serial time data suitable for processing by a 1-D CNN. Compared with 2-D CNN, 1-D CNN is a lightweight network with fewer parameters. It can reduce training complexity. In this paper, the proposed CCT classifier is designed by CNN-1D block and TCN network for more rapid implementation, where TCN is also a special type of 1-D CNN. In addition, residual connections and dropout in TCN are introduced to avoid the problem of gradient vanishing.

### 3.3. Maximum Likelihood Classifier

In order to evaluate the classification performance of the CCT classifier, the ML classifier is introduced for benchmarking purposes. It is a well-known modulation classification method, which assumes that the knowledge of channel state information and noise power is completely known for calculating the likelihood function. Usually, the ML classifier will undergo poor performance in realistic scenarios where channel parameters cannot be explicitly estimated. Moreover, it also has the problem of high computational complexity that cannot be used in real-time applications. Nevertheless, it provides an upper bound of classification performance under ideal channel conditions and can be used to evaluate other AMC algorithms [3,4].

Given received signals $\left\{r{\left(n\right)}_{n=1}^{N}\right\}$, the log-likelihood function under hypothesis $H_{j}$ of modulation scheme is given as:

(19) $$L\left(H_{j}|r_{N}\right)=\overset{N}{\underset{n=1}{\sum }}\ln\left\{\frac{1}{M_{j}}\overset{M_{j}}{\underset{m=1}{\sum }}\frac{1}{\sqrt{2\pi \sigma^{2}}}\exp\left(-\frac{1}{2\sigma^{2}}\|r_{n}-h*s_{jm}{\|}^{2}\right)\right\}$$

The classification decision of different modulation schemes is to choose the hypothesis with the largest value of the log-likelihood function, i.e.,

(20) $$H_{j}^{*}=\underset{H_{j}}{\arg\;\max}L(H_{j}|r_{N})$$

## 4. Results

In this Section, we compare the classification performance of the most commonly used features for FB-AMC problems using the CCT classifier under different channels through computer simulations. The proposed CCT classifier was executed using the deep neural network library Tensorflow2.4. The candidate set of modulation schemes M1 = {BPSK, QPSK, 8-PSK} was considered for MPSK and set M2 = {4QAM, 16QAM, 64QAM} was considered for MQAM. To evaluate the performance of the CCT classifier, we also compared it with the ideal ML classifier for different features under various channels.

### 4.1. Dataset Generation

The training, validation, and test datasets for the CCT classifier were generated using matlab2017a, which consisted of set M1 {2PSK, 4PSK, 8PSK} and set M2 {4QAM, 16QAM, 64QAM}. The numbers of received signals for each modulation scheme used were 128, 256, and 512, respectively. The range of SNR used was 0 dB to 20 dB with a step size of 1 dB. Considering the AWGN channel first, the received signals were separated into real parts and image parts and then represented by (14) to get the raw IQ dataset. Datasets of the other four features were generated by calculating received signals, according to Section 2.2.1–2.2.4. All features were rearranged using (14) and labeled with their actual modulation scheme. For each SNR of one modulation scheme, 1000 realizations were generated for received signals of different lengths. The training data under each candidate set were randomly shuffled to form a new dataset, which was further divided into the training dataset (80%) and the validation dataset (20%) for training the CCT network. Additionally, test datasets including 1000 realizations of signals were generated to evaluate the performance of the pre-trained CCT for each signal length under each SNR.

For other cases, e.g., phase offset, frequency offset, SNR errors, flat-fading channel, non-Gaussian channel, training, validation, and test, datasets were generated similarly.

### 4.2. Gaussian Channel

First, we made a comparative study about the different features for the FB-AMC problem under the Gaussian channel. Assume that the knowledge of received signals, including phase offset, frequency offset, and SNR, were all known. Simulations were performed for modulation schemes’ set M1 and M2, respectively. After generating the training data, the CCT classifier was used to learn the high-level representations from the datasets to accomplish modulation classification.

**Figure 7:**
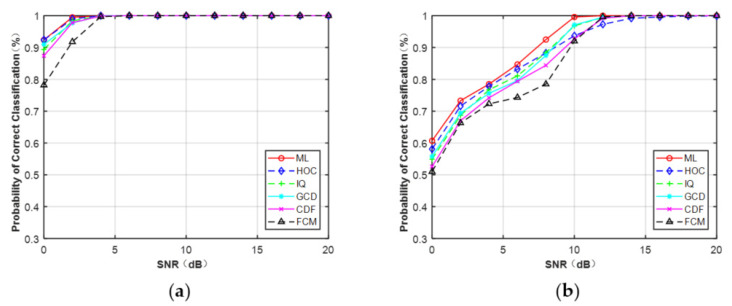
Probability of correct classification under AWGN (N = 512). (**a**) MPSK. (**b**) MQAM.

Figure 7a,b depicts the curves of the correct probability versus SNR for MPSK and MQAM when N = 512. It can be observed that, compared with the CCT classifier on the five features: HOC, IQ, GCD, CDF, and FCM, ML provided the optimal performance. However, experiments showed the AMC algorithm based on features and deep learning was faster than the ML classifier. It can be expected that the ML classifier assumed that the knowledge of the channel was fully known, which will consume more time. In addition, for each feature (N = 512), MPSK signals were identified without any error when SNR > 4 dB but for MQAM signals when SNR > 16 dB.

The overall average correct classification probability of different features for MPSK and MQAM with signal samples’ lengths of N = 128, 256, and 512 are listed in Table 3. The average probability of each feature was obtained by averaging the classification accuracy of the three PSK/QAM signals. It was clear that the performance of each feature increased as the signal length increased. Among these five features, the features of HOC, IQ, and GCD gave excellent performance, very close to that of the ideal ML classifier. Compared with the former features, CDF showed a similar performance for PSK signals but had a lower probability for classifying MQAM. FCM obtained the worst performance among the five features. The average performance difference between FCM and the ML classifier was about 2.0% for the MQAM when N = 128. As the length of signal samples increased, the performance difference began to decrease, reaching 1.4% approximately when N = 512.

Table 4 lists the average correct classification probability of BPSK, QPSK, and 8PSK at N = 128 and 4QAM, 16QAM, and 64QAM at N = 512 under Gaussian channel, respectively. It shows the classification accuracy of each modulation scheme. It is observed that all the features achieved excellent performance for BPSK and a similar performance for 4QAM. It means that BPSK was classified by all the features when SNR was set from 0 to 20 dB. All the features showed a better classification accuracy for 64QAM than 16QAM when N = 512. Among the different features, FCM showed the worst performance for QPSK, 8PSK, 4QAM, 16QAM, and 64QAM, similar to Table 3.

**Figure 8:**
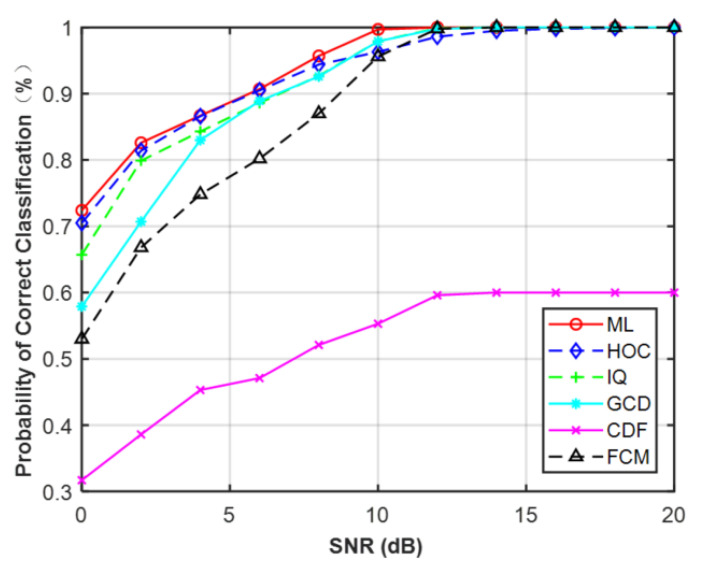
Probability of correct classification of PSK and QAM under AWGN versus SNR.

In this part, we discuss the classification performance of a more complex modulation set to verify the adaptability of the CCT classifier, including five modulation schemes, namely, 2PSK, 4PSK, 8PSK, 16QAM, and 64QAM. In Section 2.2.4, we know that CDF cannot be used to identify a set of modulation schemes, including both MPSK and MQAM, because these two types of signals have different decision statistics for generating the CDF. Therefore, the four features (HOC, IQ, GCD, and FCM) other than CDF were used to compare the performance of AMC under the Gaussian channel. Figure 8 reveals that the classification accuracy of the four features was similar to that shown in Figure 7b at N = 512. The performance of HOC was close to the ML classifier and was better than the other four features when SNR < 9 dB. However, the performance degraded in the high region of SNR. The results showed that combining more features may improve the performance of FB-AMC.

### 4.3. Estimation Errors

**Figure 9:**
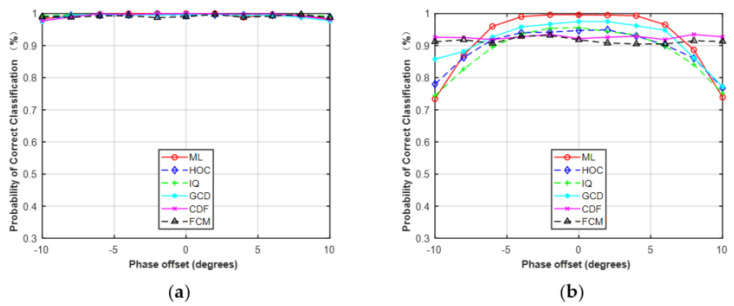
Probability of correct classification versus phase offset. (**a**) MPSK (SNR = 6, N = 128). (**b**) MQAM (SNR = 10, N = 512).

**Figure 10:**
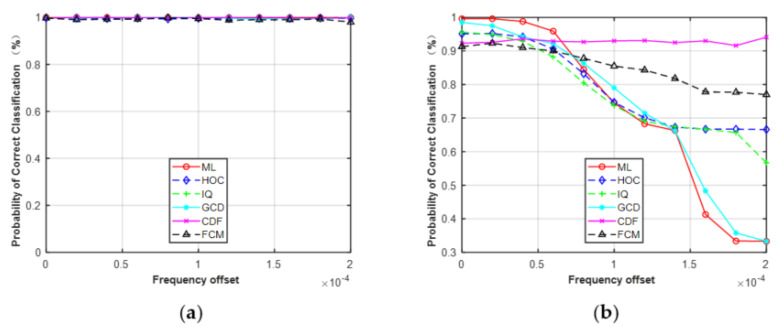
Probability of correct classification versus frequency offset. (**a**) MPSK (SNR = 6, N = 128). (**b**) MQAM (SNR = 10, N = 512).

**Figure 11:**
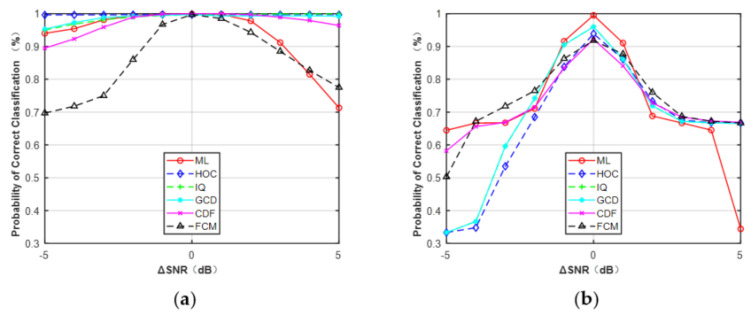
Probability of correct classification versus SNR errors. (**a**) MPSK (SNR = 6, N = 128). (**b**) MQAM (SNR = 10, N = 512).

Simulations in Section 4.2 were performed under the Gaussian channel, assuming that the CSI and noise variance were perfectly known. However, it is impossible to acquire all the channel knowledge in practice. The estimation errors of channel parameters are always inevitable. In this subsection, several simulations were performed with three estimation errors: phase offset, frequency offset, and SNR errors. To evaluate the influence of estimation errors, we regenerated the test datasets for each feature under the Gaussian channel, which were affected by the estimation errors. Then, the test data were fed into the trained CCT to evaluate the classification performance. In the simulations, the SNR and signal length was fixed at 6 dB, 128 for MPSK and 10 dB, 512 for MQAM, respectively. The simulation results are presented in Figure 9, Figure 10 and Figure 11.

In the first simulation, we only considered the estimation error of phase offset with the assumption that the carrier frequency and SNR were perfectly matched. The range of phase offset in the simulations was set from −10° to 10° with a step size of 2. The classification performance with phase offset is shown in Figure 9. For MPSK, it was obvious that the performance of these five features was robust to phase offset. For MQAM, except for CDF and FCM, the performance of the other features decreased as the phase offset increased, but the performance degradation was very small when the phase offset was set between −5° and 5°. Among the five features, CDF and FCM were robust against phase offset for MQAM.

Second, we considered the effect of frequency offset while assuming the phase offset and SNR were perfectly matched. The range of frequency offset was considered from 0 to 2 × 10^−4^ with a step size of 2 × 10^−5^. As shown in Figure 10, it can be seen that the performance of the five features was robust to the given frequency offset for MPSK. On the contrary, the classification performance of MQAM decreased as frequency offset increased, except for CDF. The reason is the same as the first simulation. When the frequency offset was > 1.6 × 10^−4^, the gap between these curves increased, especially for feature GCD and the ML classifier. The correct classification probability of ML and GCD was about 0.33 under the given frequency offset of 1.6 × 10^−4^, while the HOC remained at about 0.68 and the FCM was maintained at about 0.78.

In the third simulation, the effect of SNR errors was investigated with the assumption that the phase offset and frequency offset were all perfectly estimated. The results are shown in Figure 11, assuming that the range of SNR error was from −5 to 5 dB. It can be observed that features HOC, IQ, GCD, and CDF were less sensitive to SNR errors than FCM and the ML classifier for MPSK. However, for MQAM, all these features were sensitive to SNR errors. To avoid this problem, one feasible approach is to generate more training data with a smaller step size of SNR (e.g., 0.5 dB, 0.2 dB, or 0.1 dB), but at the expense of the overall training time.

### 4.4. Non-Gaussian Channel

Experimental results showed that most radio channels experienced non-Gaussian noise, which caused some AMC algorithms under the assumption of Gaussian noise to be no longer effective [47,48,49]. In this part, we consider the study of FB-AMC in the presence of non-Gaussian noise to look for some effective features. The same as in [47], the number of terms N in the Gaussian mixture model was set to 2. The proportion of the first term and the second term was denoted as $\lambda_{0}=0.9$, $\lambda_{1}=0.1$ with their variance denoted as $\sigma_{0}^{2}$ and $\sigma_{1}^{2}$ ($\sigma_{1}^{2}/\sigma_{0}^{2}=100$), respectively. The generating of training data under the non-Gaussian channel was similar to that under the Gaussian channel.

**Figure 12:**
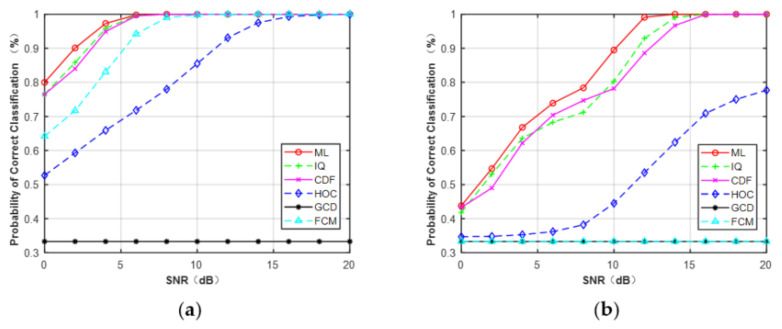
Probability of correct classification under non-Gaussian channel (N = 128). (**a**) MPSK. (**b**) MQAM.

In order to compare the classification performance of the extracted features under a non-Gaussian channel, we introduced the ideal ML classifier for benchmarking purposes. The ML classifier assumed that the parameters N, $\sigma_{0}^{2}$, $\sigma_{1}^{2}$, $\lambda_{0}$, and $\lambda_{1}$ were all known. The final form of classification decision is shown in (21). Figure 12 illustrates the simulation results when N = 128. It is clear that IQ data and CDF were useful features for MPSK and MQAM under non-Gaussian channel and achieved better performance than HOC. The GCD and FCM were invalid features for MQAM when N = 128.

(21) $$\hat{H}=\underset{H_{m}}{\;\arg\;\max}\overset{N}{\underset{n=1}{\sum }}\ln\left\{\frac{1}{N_{m}}\overset{N_{m}}{\underset{i=1}{\sum }}\overset{N}{\underset{n=1}{\sum }}\left\{\frac{\lambda_{n}^{m}}{2\pi {\left(\sigma_{n}^{m}\right)}^{2}}\exp\left(-\frac{{\left|r\left[k\right]-\alpha^{m}s_{mi}\left[k\right]\right|}^{2}}{2{\left(\sigma_{n}^{m}\right)}^{2}}\right)\right\}\right\}$$

Table 5 lists the overall average probability of correct classification of different features for MPSK and MQAM with signal lengths of N = 128, 256, 512. Like the Gaussian channel, the performance of each feature increased monotonically as the signal length increased. It is seen that the ML classifier provided the best performance for each signal length since the perfect knowledge of the channel and noise variance was already known. For MPSK, the performance of CDF was similar to IQ but better than FCM. When the signal length was short, GCD could not distinguish these two types of signals. For MQAM, the performance of CDF was worse than IQ when N = 128, but it outperformed IQ as the length of the signal increased. Additionally, FCM and GCD are features that are only valid for long signals. The performance of HOC was not satisfactory for both MPSK and MQAM signals.

### 4.5. Flat-Fading Channel

As we know, channel fading can cause severe degradation in the modulation classification performance when the CSI is not estimated. In this subsection, we consider the performance comparison of modulation classification in a flat-fading channel. Therefore, the α and $\theta_{0}$ remained unchanged during one observation period [37,45]. The PDF of α was assumed to be Rayleigh distribution, given by (22). Training data generation for the CCT classifier was the same as the previous simulations.

(22) $$p\left(\alpha \right)=\frac{2\alpha }{\sigma_{\alpha }^{2}}\exp\left(-\frac{\alpha^{2}}{\sigma_{\alpha }^{2}}\right),\;\alpha >0$$

**Figure 13:**
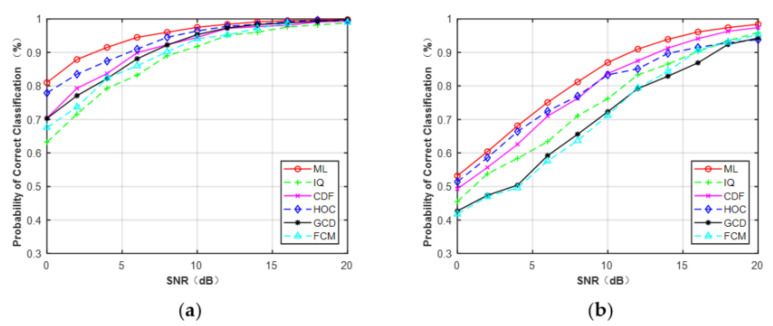
Probability of correct classification under flat-fading channel (N = 512). (**a**) MPSK. (**b**) MQAM.

Additionally, the ML classifier with perfect knowledge of CSI was introduced as a benchmarking classifier. Simulation results at N = 512 are shown in Figure 13. It is seen that ML provided better performance than CCT classifier on the different features of each SNR. The average performance of ML was about 95.2% for MPSK and 82.5% for MQAM. Compared with Gaussian channel, the average performance degraded about 4.1% and 7.4% for MPSK and MQAM, respectively. Therefore, it proved that channel fading can cause modulation classification performance to decrease.

Under flat-fading channel, the overall average correct classification probability of different features for MPSK and MQAM with signal lengths of N = 128, 256, 512 are listed in Table 6. It can be observed that HOC obtained the best performance compared to the other features for MPSK. However, it was worse than CDF for MQAM. It was noted that the performance difference between HOC and CDF decreased as the signal length increased. When N = 512, HOC and CDF had a very competitive performance. In addition, GCD could not distinguish these two types of signals when the signal samples’ length was short, which is the same as the non-Gaussian channel. The raw IQ data showed the worst performance compared to other features for MPSK at N = 512, but they were better than GCD and FCM for MQAM.

### 4.6. Transfer Learning

In this study, more than 100 experiments were performed for different channels, features, and signal lengths. In fact, with the help of transfer learning [56], we did not need to train every DNN from the beginning. Transfer learning is an effective strategy to use a pre-trained DNN on other similar datasets. It can use the fine-tuning weights trained on the former dataset as the initial weights for the new task. Then the new task will be trained based on prior knowledge or experiences instead of random parameters, which further reduce the training time.

**Figure 14:**
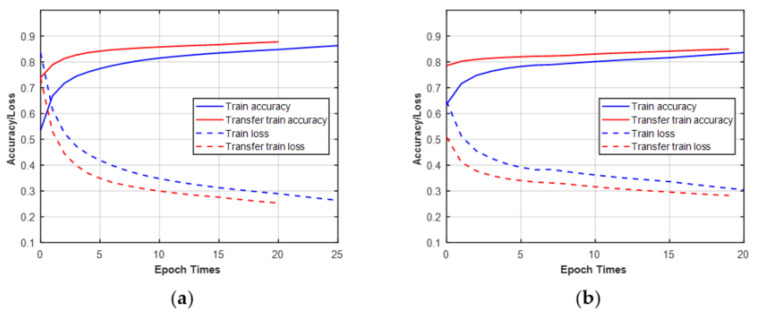
Training/validation curves of accuracy and loss (blue lines: without transfer learning; red lines: with transfer learning. (**a**) Training on MPSK transfer to MQAM at N = 512; (**b**) training under AWGN transfer to non-Gaussian for MQAM at N = 512.

In this part, we give two examples of using transfer learning to train new tasks. First, the CCT classifier was trained on raw IQ datasets of MPSK at N = 512. Then, the pre-trained network was transferred to train the raw IQ datasets of MQAM. Second, the CCT classifier was trained on datasets of MQAM under the Gaussian channel at N = 512. Then, the pre-trained network was transferred to train the datasets of MQAM under the non-Gaussian channel. As shown in Figure 14, the ascent speed of transfer accuracy and descent speed of transfer loss were faster than the original training when the transfer learning was performed between different modulation scheme sets or channel conditions. In addition, although transfer learning helps to improve training efficiency, it cannot improve classification performance.

### 4.7. Comparison with Exiting Classifiers

To evaluate the performance of the proposed CCT classifier, we compared it with the CNN classifier proposed in [36] and the traditional K–S classifier proposed in [21], respectively.

**Figure 15:**
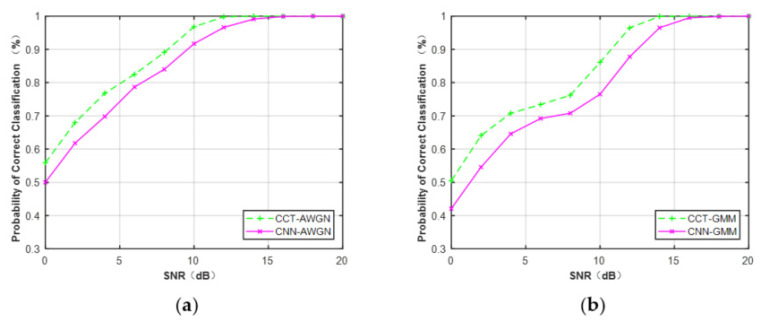
Performance comparison of MQAM based on the feature of raw IQ data (N = 512). (**a**) AWGN. (**b**) Non-Gaussian channel.

First, raw IQ data were considered as training data to evaluate the classification performance of CCT and CNN classifiers. Training data of MQAM at N = 512 under Gaussian channel and non-Gaussian channel were generated. In Figure 15, it is obvious that the CCT classifier provided better performance versus each SNR at N = 512 compared with the CNN classifier. The average performance difference between CCT and CNN classifiers was about 3.4% under the Gaussian channel and 5.1% under the non-Gaussian channel.

Table 7 lists the performance comparison of CCT and CNN classifiers for all the modulation schemes. It can be seen that the CNN and CCT classifiers achieved similar performance for MPSK. However, for MQAM, the CCT classifier provided better performance than the CNN classifier in both Gaussian and non-Gaussian channels.

**Figure 16:**
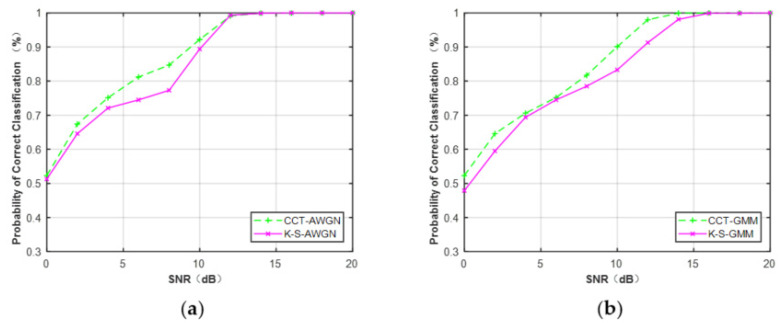
Performance comparison of MQAM based on the feature CDF (N = 512). (**a**) AWGN. (**b**) Non-Gaussian channel.

Second, we compared the performance of CCT and K–S classifiers on the feature CDF. CDFs of the MQAM signal at N = 512 under Gaussian channel and non-Gaussian channel are considered in this part. From Figure 16, it can be seen that the CCT classifier provided better performance than the K–S classifier when N = 512. The average performance difference between CCT and K–S classifiers was approximately 2.1% under the Gaussian channel and 2.7% under the non-Gaussian channel.

Table 8 lists the performance comparison of CCT and K–S classifiers for all the modulation schemes. It can be seen that the CCT classifier achieved better performance than the traditional K–S classifier for MPSK and MQAM under Gaussian and non-Gaussian channels, respectively.

## 5. Discussion

In this article, a comparative study of FB-AMC problems among the commonly used features was carried out using deep learning methods. CCT classifier based on the end-to-end deep neural network was designed to evaluate the classification performance of different features, including HOC, FCM, GCD, CDF, and raw IQ data. Extensive experiments were performed to compare the classification performance of each feature under Gaussian, non-Gaussian, and flat-fading channels.

Simulation results showed that all the features can be used to deal with the problem of FB-AMC under Gaussian channel. HOC, IQ, and GCD showed superior performance, close to the ideal ML classifier in the different features. For MPSK, the average performance difference between these features and the ML classifier was less than 0.5% at N = 512. For MQAM, the difference was approximately 2.0% at N = 512. Compared with HOC and raw IQ data, the classification accuracy of CDF and FCM was slightly worse, but they were less sensitive to the given phase offset (−10°–10°) or frequency offset (0–2 × 10^−4^) under the Gaussian channel at SNR = 6 dB. For example, when the frequency offset was >1.6 × 10^−4^, the correct classification probability of ML and GCD was about 0.33, while the HOC remained at about 0.68 and the FCM was maintained at about 0.78. The reason is that the decision statistic for calculating CDFs is the magnitude or phase of received signals, which was not affected by the given phase offset. Similarly, the clusters derived from the FCM algorithm will remain unchanged even if received signals take a rotation caused by phase offset or frequency offset.

However, only CDF and raw IQ data were the feasible features for both MPSK and MQAM in the non-Gaussian channel. The classification rate of the remaining features became lower when non-Gaussian noise was introduced. It was noted that the raw IQ data were an effective and robust feature under these different channel conditions since the raw IQ data contained all the amplitude and phase information of the received signals. In practice, it is easy to get the raw IQ data under various channels. Therefore, they provide an effective data-driven approach for AMC problems when encountering non-ideal channel conditions. In addition, GCD could not distinguish MPSK or MQAM signals when the signal length was short under non-Gaussian and flat-fading channels. However, as the length of signals increased, GCD became effective. Lastly, HOC and CDF had very competitive performance under the flat-fading channel.

The comparison results showed that the performance of the CCT classifier was better than that of the CNN classifier proposed in [36], about 3.4% in Gaussian channel and 5.1% in non-Gaussian channel when N = 512. One of the reasons is that the CCT classifier took more filter kernels and became deeper than the CNN classifier. From the literature, it is well known that a deeper DNN will learn more high-level information, which is beneficial to the further classification task. In this study, the proposed CCT classifier composed of CNN-2D, CNN-1D, and TCN was deeper than the CNN classifier. Therefore, it could learn more discriminative information from raw IQ data and obtain better performance than the CNN classifier. We also demonstrated that the CCT classifier achieved better performance than the traditional K–S classifier. The average performance difference between CCT and the K–S classifier was about 2.1% under the Gaussian channel and 2.7% under the non-Gaussian channel for MQAM when N = 512. This is because the CCT classifier could learn the distinct information within all the test points, while the K–S classifier only considered one test point with the maximum distance.

It should be noted that the phase offset, frequency offset, and SNR errors were limited to a fixed range when performing the simulations in Section 4.3. The phase offset, frequency offset, and SNR errors were limited to −10°–10°, 0–2 × 10^−4^, and −5–5 dB, respectively. The results in Figure 9, Figure 10 and Figure 11 were based on these assumptions. As shown in Figure 9 and Figure 10, the performance of the five features was robust to the given phase offset and frequency offset for MPSK. Here, we increased the estimation errors to evaluate the effect on classification performance. The phase offset was set to 10°–40° and the frequency offset was set to 2 × 10^−4^–12 × 10^−4^.

**Figure 17:**
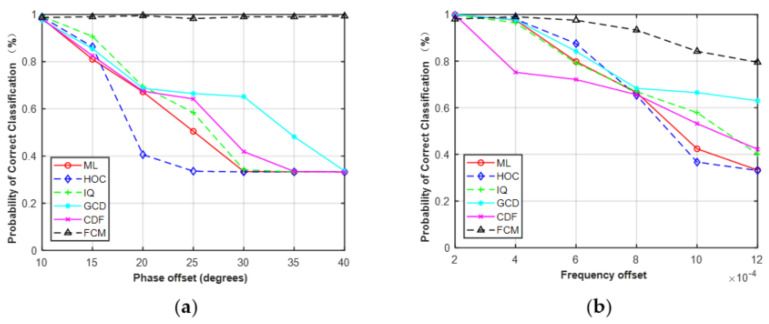
Probability of correct classification of MPSK (SNR = 6, N = 128) versus phase offset (**a**) and frequency offset (**b**).

As shown in Figure 17, the performance of HOC, IQ, GCD, CDF, and ML began to decrease as the estimation errors increased. However, FCM was robust to the given phase offset and less sensitive to frequency offset. It should be noted that, in practical applications, the phase, frequency, and SNR of received signals need to be estimated before AMC. Therefore, it is necessary to accurately estimate the parameters of the received signals, which helps to improve the classification performance of AMC.

Inspired by the development of deep learning and the results of previous simulations, we can observe some work that needs to be studied in the future. Our further research includes data-driven AMC, dataset augmentation for AMC, deep clustering algorithms for AMC, and joint channel estimation and AMC based on deep learning.

1. Develop data-driven modulation classification under various channels based on deep neural networks. In this study, we found the raw IQ data are an effective feature for different channels. Therefore, we will extend it to other complex communication environments, such as MIMO channels, frequency-selective channels, and time-variant channels [57]. More complex deep networks will be considered, e.g., Resnet, Densenet, and Inception_resnet.
2. Dataset augmentation can enhance the performance and generalizability of DNNs [43]. Given a training dataset of communication signals, some operations such as adding noise, rotating, and rescaling can be introduced to enlarge the dataset. Another feasible work is to look for some generative models such as VAE (Variational Autoencoder) and GAN to augment the training data [43]. We proved that generating training data with a smaller step size of SNR (e.g., 0.1 dB) can improve the classification performance.
3. Deep clustering algorithms, e.g., deep k-means and deep fuzzy-c-means, can be introduced to improve classification performance. Clustering is a data-driven algorithm, which relies on the quality of data points’ representations [58,59]. Deep clustering algorithms can be used to learn more distinct clustering features of data points through deep neural networks, (e.g., as AE, CNN, LSTM), which will further improve the performance of AMC.
4. Deep learning models have been used to address communication channel estimation and achieved better performance [60]. We believe that combining channel estimation and AMC via deep learning is a promising method.

## 6. Conclusions

In this study, a new CCT classifier combining CCNs and TCN was designed to address FB-AMC problems by extracting high-level information from different features (HOC, GCD, FCM, CDF, and raw IQ data). All features except GCD were converted to the same format to meet the input format of the proposed CCT classifier, aiming at designing a general neural network for FB-AMC problems. This study shows that the CCT classifier is a robust architecture that can be used to evaluate the classification accuracy of extracted features under three different channels. The comparison results also show that the CCT classifier is superior to existing CNN and K–S test classifiers.

## Figures and Tables

**Table 1:** Theoretical values of HOCs for some modulation schemes with unity energy.

Mod/HOC	$C_{40}$	$C_{41}$	$C_{42}$	$C_{60}$	$C_{61}$	$C_{62}$	$C_{63}$
BPSK	−2	−2	−2	16	16	16	16
QPSK	1	0	−1	0	−4	0	4
8PSK	0	0	−1	0	0	0	4
4QAM	1	0	−1	0	−4	0	4
16QAM	−0.68	0	−0.68	0	2.08	0	2.08
64QAM	−0.619	0	−0.619	0	1.797	0	1.797

**Table 2:** The formats of different features for AMC.

Features	Description	Shape
HOCs	$C_{20},\;C_{21},C_{40},C_{41},\;C_{42},\;C_{60},\;C_{61},\;C_{62},\;C_{63}$	1 × 9
GCD	images	64 × 64 × 3
FCM	$(T_{2},\;T_{4},T_{8}$$)\;\mathrm{or}\;(T_{4},\;T_{16},T_{64}$)	1 × 3
CDF	series data	1 × N
Raw IQ	IQ data	2 × N

**Table 3:** Average probability of correct classification of different features under Gaussian channel at N = 128, 256, and 512.

	ML	HOC	IQ	GCD	CDF	FCM
PSKs (N = 128)	0.975	0.970	0.971	0.971	0.968	0.942
PSKs (N = 256)	0.985	0.983	0.983	0.979	0.980	0.957
PSKs (N = 512)	0.993	0.992	0.989	0.987	0.986	0.972
QAMs (N = 128)	0.846	0.811	0.816	0.820	0.798	0.779
QAMs (N = 256)	0.871	0.847	0.855	0.842	0.836	0.820
QAMs (N = 512)	0.899	0.880	0.878	0.877	0.865	0.849

**Table 4:** Average correct classification probability of each modulation scheme of different features under Gaussian channel.

	ML	HOC	IQ	GCD	CDF	FCM
BPSK (N = 128)	1.000	1.000	0.997	1.000	0.999	1.000
QPSK (N = 128)	0.965	0.952	0.968	0.948	0.960	0.893
8PSK (N = 128)	0.962	0.957	0.949	0.965	0.946	0.933
4QAM (N = 512)	0.986	0.980	0.975	0.975	0.970	0.967
16QAM (N = 512)	0.849	0.826	0.809	0.803	0.810	0.778
64QAM (N = 512)	0.862	0.834	0.852	0.854	0.816	0.801

**Table 5:** Average probability of correct classification of different features under non-Gaussian channel at N = 128, 256, and 512.

	ML	HOC	IQ	GCD	CDF	FCM
PSKs (N = 128)	0.970	0.821	0.961	0.333	0.959	0.920
PSKs (N = 256)	0.980	0.831	0.973	0.333	0.971	0.954
PSKs (N = 512)	0.989	0.841	0.981	0.930	0.983	0.971
QAMs (N = 128)	0.824	0.512	0.791	0.333	0.784	0.333
QAMs (N = 256)	0.843	0.511	0.808	0.333	0.816	0.766
QAMs (N = 512)	0.854	0.520	0.834	0.779	0.848	0.787

**Table 6:** Average probability of correct classification of different features under flat-fading channel at N = 128, 256, and 512.

	ML	HOC	IQ	GCD	CDF	FCM
PSKs (N = 128)	0.927	0.896	0.843	0.333	0.870	0.856
PSKs (N = 256)	0.941	0.916	0.855	0.333	0.893	0.873
PSKs (N = 512)	0.952	0.932	0.876	0.908	0.910	0.894
QAMs (N = 128)	0.770	0.692	0.703	0.333	0.723	0.667
QAMs (N = 256)	0.798	0.729	0.728	0.679	0.757	0.683
QAMs (N = 512)	0.825	0.784	0.746	0.703	0.786	0.703

**Table 7:** Performance comparison of CNN and CCT of different modulation schemes under AWGN and non-Gaussian channel at N = 128, 256, and 512.

	CNN (AWGN)	CCT (AWGN)	CNN (GMM)	CCT (GMM)
PSKs (N = 128)	0.970	0.971	0.949	0.961
PSKs (N = 256)	0.980	0.983	0.965	0.973
PSKs (N = 512)	0.988	0.989	0.976	0.981
QAMs (N = 128)	0.810	0.816	0.750	0.791
QAMs (N = 256)	0.824	0.855	0.778	0.808
QAMs (N = 512)	0.846	0.878	0.783	0.834

**Table 8:** Performance comparison of K–S and CCT of different modulation schemes under AWGN and non-Gaussian channel at N = 128, 256, and 512.

	K-S (AWGN)	CCT (AWGN)	K-S (GMM)	CCT (GMM)
PSKs (N = 128)	0.936	0.968	0.921	0.959
PSKs (N = 256)	0.954	0.980	0.943	0.971
PSKs (N = 512)	0.966	0.986	0.956	0.983
QAMs (N = 128)	0.787	0.798	0.743	0.784
QAMs (N = 256)	0.815	0.836	0.782	0.816
QAMs (N = 512)	0.844	0.865	0.820	0.848

## Data Availability

Not applicable.
